# Effects of a Single Dose of Parecoxib on Inflammatory Response and Ischemic Tubular Injury Caused by Hemorrhagic Shock in Rats

**DOI:** 10.1155/2018/8375746

**Published:** 2018-02-04

**Authors:** Mariana Takaku, Andre Carnevali da Silva, Nathalie Izumi Iritsu, Pedro Thadeu Galvao Vianna, Yara Marcondes Machado Castiglia

**Affiliations:** ^1^Hospital Regional do Câncer de Presidente Prudente, Presidente Prudente, SP, Brazil; ^2^Hospital Santa Casa de Misericórdia de Vitória, Vitória, ES, Brazil; ^3^A.C.Camargo Cancer Center, São Paulo, SP, Brazil; ^4^Department of Anesthesiology, Botucatu School of Medicine, São Paulo State University, Botucatu, SP, Brazil

## Abstract

Parecoxib, a selective COX-2 inhibitor, is used to improve analgesia in postoperative procedures. Here we evaluated whether pretreatment with a single dose of parecoxib affects the function, cell injury, and inflammatory response of the kidney of rats subjected to acute hemorrhage. Inflammatory response was determined according to serum and renal tissue cytokine levels (IL-1*α*, IL-1*β*, IL-6, IL-10, and TNF-*α*). Forty-four adult Wistar rats anesthetized with sevoflurane were randomized into four groups: placebo/no hemorrhage (Plc/NH); parecoxib/no hemorrhage (Pcx/NH); placebo/hemorrhage (Plc/H); and parecoxib/hemorrhage (Pcx/H). Pcx groups received a single dose of intravenous parecoxib while Plc groups received a single dose of placebo (isotonic saline). Animals in hemorrhage groups underwent bleeding of 30% of blood volume. Renal function and renal histology were then evaluated. Plc/H showed the highest serum levels of cytokines, suggesting that pretreatment with parecoxib reduced the inflammatory response in rats subjected to hemorrhage. No difference in tissue cytokine levels between groups was observed. Plc/H showed higher percentage of tubular dilation and degeneration, indicating that parecoxib inhibited tubular injury resulting from renal hypoperfusion. Our findings indicate that pretreatment with a single dose of parecoxib reduced the inflammatory response and tubular renal injury without altering renal function in rats undergoing acute hemorrhage.

## 1. Introduction

Nonsteroidal anti-inflammatory drugs (NSAIDs) are among the most widely prescribed therapeutic agents for reducing acute pain and are often used to improve analgesia and reduce opioid consumption, accelerating recovery after surgery [1–3].

Conventional NSAIDs block both cyclooxygenase (COX) enzyme isoforms, COX-1 and COX-2. The potential adverse effects of cyclooxygenase-1 inhibition, such as gastric ulceration and bleeding, changes in hemostasis, and adverse renal effects, have limited the use of NSAIDs in many surgical patients [3, 4]. Thus, novel NSAIDs that selectively inhibit COX-2 have been developed to reduce these adverse effects, with similar efficacy to that of conventional NSAIDs [5]. Parecoxib is a COX-2 inhibitor effective and safe in postoperative analgesia of noncardiac surgery [2, 6, 7].

Inflammation plays a major role in the pathophysiology of acute ischemic renal injury [8, 9]. The renal ischemia promotes endothelial and tubular cell injury, release of inflammatory mediators, such as cytokines and chemokines, and leukocyte infiltration into the renal tissue [9]. The levels of proinflammatory cytokines IL-1*α*, IL-1*β*, IL-6, and TNF-*α* are higher in ischemic renal injury and these cytokines are involved in different mechanisms of renal tissue injury. By contrast, IL-10 is an anti-inflammatory cytokine that inhibits cytotoxicity and inflammation pathways and protects from ischemic renal injury [8–11].

Some studies suggest that selective COX-2 inhibitors worsen renal function in hypoperfusion and ischemia events [12–14]. Thus, it is still controversial whether parecoxib would be a good option for analgesia in such cases. Reduction of the inflammatory response and prevention of organ injury are a major concern in the treatment of pain in these cases.

The aim of this study was to evaluate whether pretreatment with a single dose of parecoxib affects renal function, renal tissue injury, and inflammatory response in rats submitted to acute hemorrhage.

## 2. Methods

The study was approved by the Animal Experimentation Ethics Commission, College of Medicine of Botucatu, UNESP, São Paulo, Brazil. After being anesthetized by sevoflurane, 44 adult male Wistar rats (20 to 24 weeks old) were randomly allocated into four groups as described below:

- placebo/no hemorrhage (Plc/NH): rats kept normovolemic, receiving 0.9% saline (2 ml·kg^−1^ IV) after anesthesia (*n* = 11);
- parecoxib/no hemorrhage (Pcx/NH): rats kept normovolemic, treated with parecoxib (20 mg·kg^−1^ at 2 ml·kg^−1^ IV) after anesthesia (*n* = 11);
- placebo/hemorrhage (Plc/H): rats submitted to hemorrhage of 30% of the blood volume, receiving 0.9% saline (2 ml·kg^−1^ IV) after anesthesia and 30 minutes before bleeding (*n* = 11);
- parecoxib/hemorrhage (Pcx/H): rats submitted to hemorrhage of 30% of the blood volume, treated with parecoxib (20 mg·kg^−1^ at 2 ml·kg^−1^ IV) after anesthesia and 30 minutes before bleeding (*n* = 11).

The separation of animals in groups was performed at the beginning of the experiment using a computer program that randomly ordered a sequence of steps to be followed. None of the animals died or had to be excluded from the study.

Rats were submitted to inhalation anesthesia with 2,5% sevoflurane (vaporizer, Ohmeda, USA) administered by a mask nonrebreathing system under spontaneous respiration. Rectal temperature (*T*) was monitored by a rectal thermometer and maintained between 37°C and 38°C with a thermal blanket. The internal right jugular vein was dissected and cannulated with 24GA venocath to maintain infusion of Ringer lactate solution (RL) (5 mL·kg^−1^·h^−1^) [15, 16] and to inject sodium para-aminohippurate (PAH), sodium iothalamate (IOT), and parecoxib. Next, the left carotid artery was dissected and cannulated with a 24GA venocath to monitor mean arterial pressure (MAP) through the transducer of a Datex Engstrom recorder (Finland). Immediately after internal jugular vein catheterization, all groups received 1 mg prime solution of PAH (20%, Sigma) and 0.5 mg IOT (70%, Mallinckrodt) in 0.5 mL 0.9% NaCl solution. Then, continuous infusion was initiated for both agents: 1 mg·h^−1^ PAH and 0.25 mg·h^−1^ IOT in 0.9% NaCI solution until the end of the experiment [17] using an Anne® infusion pump (Abbott, USA). Immediately after prime solution injection, rats received a bolus (2 mL·kg^−1^) of saline or parecoxib according to their distribution by groups. The solution was prepared by a technician unaware of the study and the procedure was performed blindly by the researcher. The selected dose of parecoxib was based on previous studies using this drug in models of ischemia-reperfusion in rats [13].

Bleeding was initiated 30 minutes after placebo/parecoxib infusion (*T*30′) in Plc/H and Pcx/H groups via carotid artery in three steps, 10 minutes apart, which corresponded to a total blood drawing of 30% of the blood volume, ending at *T*50′ time point. Blood volume was estimated as 6% of total animal body weight [15, 16].

For all groups, the hematocrit and concentrations of PAH, IOT, and serum cytokines were determined in the arterial blood collected 30 minutes after the last bleeding time point in hemorrhage groups, and 80 minutes after IOT/PAH primes and placebo/parecoxib bolus in nonhemorrhage groups (*T*80′, end time point). At this moment, rats underwent laparotomy for bilateral nephrectomy and then were sacrificed by IV injection of sodium pentobarbital. A drop of blood was also collected after venous dissection in all groups to determine the initial hematocrit (*T*0, control time point). PAM was measured at *T*0, *T*30′ (onset of bleeding in hemorrhage groups), *T*50′ (end of bleeding in hemorrhage groups), and *T*80′ moments.

Blood samples for subsequent analysis of serum cytokines were centrifuged for 10 minutes at 1200*g* at 4°C. Sera were frozen and stored at −70°C. The experimental model followed methodology adequate for measuring renal clearance in small rodents with reduced urine volume after hemorrhage [17]. IOT and PAH concentrations (mg·mL^−1^) were obtained by high performance liquid chromatography (HPLC), and clearances (*C*) of IOT and PAH (*C*_IOT_ and *C*_PAH_) was calculated according to the Fick principle:

(1) Cml·min−1=constant infusion rate mg·min−1mean arterial concentration mg·mL−1

*C*_PAH_ estimated the effective renal plasma flow (ERFP), and *C*_IOT_ estimated the glomerular filtration rate (GFR). Filtration fraction (FF) was defined as GFR/ERFP (*C*_IOT_/*C*_PAH_), renal blood flow (RBF) as ERFP/(1 − Ht), and renal vascular resistance (RVR) as MAP/RBF.

The analysis of hematocrit was performed by microhematocrit method (Centremicro, Fanem, Brazil) and the result was expressed as a percentage.

Serum concentrations of IL-1*α*, IL-1*β*, IL-6, IL-10, and TNF-*α* were determined using kits for ELISA (R&D Systems: Minneapolis, MN, USA, and BD Sciences: San Diego, CA, USA) and the result was expressed as picogram per milliliter of serum.

Both removed kidneys were longitudinally sectioned into two halves. One half of each kidney was taken for histological preparation. The other remaining halves were macerated and homogenized with 4 mL of PBS (sterile phosphate buffer, pH = 7.4, Gibco, Invitrogen). The samples were centrifuged for 15 minutes at 3000*g* at 4°C, obtaining the supernatant renal proteins, which were frozen and stored at −70°C for later determination of tissue cytokine levels. The dosages of IL-1*α*, IL-1*β*, IL-6, IL-10, and TNF-*α* were determined in the supernatant kidney by ELISA and the result was expressed as picogram per milligram of protein.

The remaining kidney halves separated for histological analysis were placed in Duboscq-Brazil solution (120 mL formol, 30 mL acetic acid, and 2 g picric acid) for 24 hours and then stored in 70% ethanol. Slices with fragments from both kidneys were prepared and colored with hematoxylin/eosin. The histological slides were evaluated for the presence of vascular dilation, vascular congestion, tubular dilation, tubular degeneration, and tubular necrosis. Scores were attributed for each of these histological changes, corresponding to severity of injury: zero (0), absence of lesion; one (1), discreet lesions; two (2), moderate lesions; and three (3), intense lesions [15]. The histological analysis was performed blindly by a pathologist.

The sample size was determined based on mean values and standard deviations observed in the literature for GFR, considering an average difference between the mean of 0.017 mL·min^−1^ and a standard deviation of 0.012, statistical power of 90%, and 5% level of significance, which resulted in 11 animals per group [13].

### 2.1. Statistical Analysis

Weight was analyzed by Student's *t*-test. Attributes in the animals evaluated over time were analyzed by Profile Analysis, followed by Tukey test for the results of clearances of PAH and IOT, FF, RBF, and RVR. Histological variable values in each kidney and comparison between groups were analyzed by Chi-Square test. In all analyses, statistics were considered significant when *p* < 0.05.

## 3. Results

**Figure 1:**
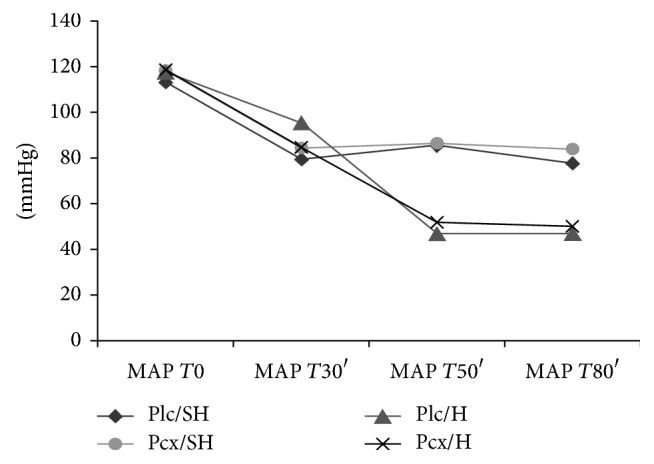
Mean arterial pressure (MAP) of the four groups of rats according to the different time points.

Weight was similar in all groups (424 g ± 65). Rectal temperature remained stable in all groups (37,6°C ± 0,4). Mean arterial pressure (MAP) of hemorrhage groups (Plc/H and Pcx/H) decreased significantly after bleeding (Figure 1) and was lower than that in nonhemorrhage groups (Plc/NH and Pcx/NH) at *T*50′ and *T*80′ time points (Figure 1). There was no difference in MAP between groups at *T*0 and *T*30′ (Figure 1).

**Figure 2:**
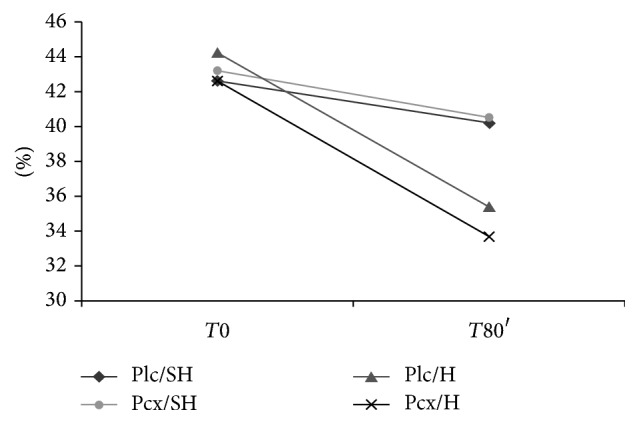
Hematocrit of the four groups of rats at *T*0 (control time point) and *T*80′ (end time point).

Hemorrhage groups (Plc/H and Pcx/H) had a significant decrease in hematocrit at *T*80′ showing lower values than non-hemorrhage groups (Plc/NH and Pcx/NH) at this time point (Figure 2).

Serum levels of IL-1*α*, IL-1*β*, IL-6, IL-10, and TNF-*α* were higher in Plc/H group (Table 1).

There was no difference among groups regarding the mean values of IL-1*α*, IL-1*β*, IL-6, IL-10, and TNF-*α* in the supernatant renal tissue (Table 2).

We observed that ERFP (*C*_PAH_) was significantly higher in Plc/NH as compared to groups Plc/H and Pcx/H (Table 3) and higher in Pcx/NH as compared to Plc/H (Table 3). GFR (*C*_IOT_) was higher in Pcx/NH compared to Plc/H (Table 3), with the other two groups showing intermediate values. FF was similar in all groups (Table 3). RVR presented values significantly higher in Plc/H than in Plc/NH (Table 3) and Pcx/NH and Pcx/H showed intermediate values.

**Figure 3:**
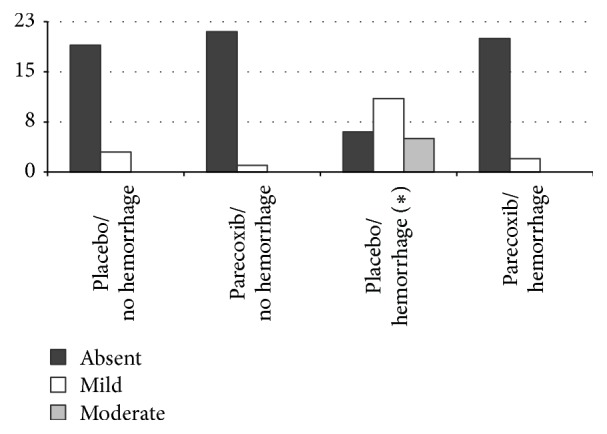
Frequency distribution of tubular dilation in the kidneys in each of the four groups of rats (^*∗*^*p* < 0.05).

**Figure 4:**
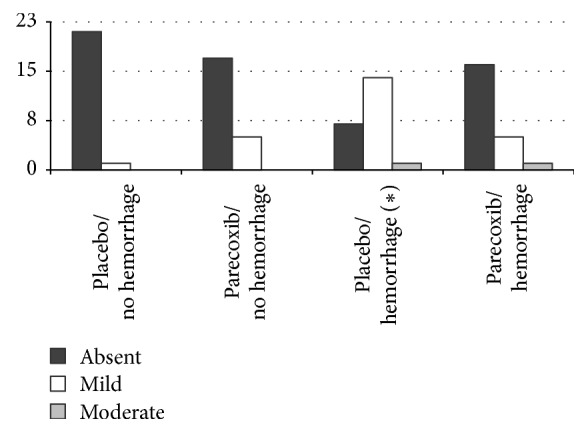
Frequency distribution of tubular degeneration in the kidneys in each of the four groups of rats (^*∗*^*p* < 0.05).

Histological analysis of the kidneys showed mild degrees of vascular congestion and vascular ectasia in all groups. The Plc/H group had more tubular dilation and tubular degeneration, both mild and moderate, than the other groups (Figures 3 and 4). There was no case of tubular necrosis in any group.

## 4. Discussion

In this study, we observed a decrease in MAP in hemorrhage groups (Plc/H and Pcx/H), due to hypovolemia. Likewise, hematocrit values of Plc/H and Pcx/H were significantly lower at end time point, if compared to control time point in the same groups or to either moment in nonhemorrhage groups. There was a slight but not significant reduction in hematocrit in nonhemorrhage groups at end time point. This may be due to hemodilution resulting from intravenous infusions during the experiment.

Ischemic acute renal injury is considered an inflammatory disease. Endothelial injury, release of inflammatory mediators, and leukocyte infiltration are the basis of renal tubular injury that occurs after ischemic events [9]. Thus, the increased levels of inflammatory mediators reflect renal injury observed in renal hypoperfusion. Ischemia of any organ increases the production of proinflammatory cytokines IL-1, IL-6, and TNF-*α* [9, 10, 18]. Plc/H had serum levels of IL-1*α*, IL-1*β*, IL-6, IL-10, and TNF-*α* higher than the other groups while Pcx/H had serum levels of cytokines similar to nonhemorrhage groups (Table 1), suggesting that parecoxib contributed to inhibiting serum production and serum release of these inflammatory mediators in hemorrhagic stress events. Feitoza et al. [19] also observed a significant reduction in levels of IL-1*β* and TNF-*α* in rats with ischemia-reperfusion renal injury pretreated with indomethacin, a nonselective NSAID. This demonstrates the potential of COX inhibitors in attenuating the inflammatory response resulting from renal ischemic injury. Pretreatment with parecoxib did not affect serum cytokines in nonhemorrhage groups, as in these cases no ischemia or even the subsequent inflammatory response occurred.

The increase in serum levels of proinflammatory cytokines IL-1*α*, IL-1*β*, IL-6, and TNF-*α* in the groups submitted to hemorrhage agrees with findings in other studies showing greater production of these inflammatory mediators in renal injury [8, 18, 20]. Despite having anti-inflammatory properties, increased serum level of IL-10 is a predictor of increased mortality in patients with acute kidney injury [21]. Here we observed increased serum levels of IL-10 in the group in which bleeding was pretreated with placebo (Table 1), revealing the anti-inflammatory response that cooccurs with hemorrhage. The increase of this cytokine was also inhibited by prior administration of parecoxib, similarly to that observed with the proinflammatory cytokines when parecoxib was given.

COX-2 expression is elevated in kidneys subjected to* ischemia*-reperfusion injury and its activation in inflammation has a negative impact on renal function [22]. Increased expression of COX-2 in the macula densa in situations of renal hypoperfusion contributes to renal vasoconstriction response, causing decline in RBF and GFR, by a tubuloglomerular* feedback* mechanism [23, 24]. Thus, selective blocking of COX-2 with parecoxib reduces the inflammatory response in kidneys subjected to ischemia due to hemorrhage and also suppresses vasoactive response in glomerular vessels resulting from tubuloglomerular* feedback*. McDonald et al. [25] observed substantial renal injury as well as increase in expression of COX-2 protein in kidney of rats subjected to hemorrhage and resuscitation. They document that the pretreated with selective COX-2 inhibitor SC58635 30 minutes before hemorrhage attenuates the rise in the serum levels of creatinine caused by hemorrhagic shock. Feitoza et al. [22] observed that pretreatment with selective COX-2 inhibitor promoted even more significant improvement in renal function in rats undergoing ischemia-reperfusion renal injury than pretreatment with nonselective NSAID. The mechanisms involved in renal protection of COX inhibitors are not fully elucidated. Previous studies have shown that COX inhibitors are able to suppress TNF-*α*, IL-6, and IL-1*β* in renal tissue after ischemia [26]. Likewise, our results show that use of parecoxib reduces the inflammatory response resulting from hemorrhage, as indicated by lower serum levels of cytokines in rats pretreated with this NSAID.

There was no difference between groups for the levels of IL-1*α*, IL-1*β*, IL-6, IL-10, and TNF-*α* obtained from the supernatant of homogenized renal tissue. This may be due to the extraction time of the kidneys during the procedure. Nephrectomy was performed 30 minutes after the end of hemorrhage, which might be an insufficient time for increasing production and release of cytokines by the kidney cells. Likewise, Kurcer et al. [27] showed that renal ischemia in rats promoted structural and functional alterations in this organ, without increasing levels of IL-1*β*, IL-6, and TNF-*α* in renal supernatant tissue. Donnahoo et al. [28] did not observe an increase in renal tissue levels of TNF-*α* after 30 and 60 minutes of renal ischemia, but rather after one hour of renal ischemia followed by one hour of reperfusion. However, after 30 minutes of renal ischemia, they found higher expression of TNF-*α* mRNA in renal tissue.

Increased serum proinflammatory cytokines after renal injury may be due to several factors, such as higher production by lymphocytes, monocytes, macrophages, endothelial cells, and fibroblasts and by renal tubular epithelial cells and mesangial cells [9, 18, 21]. The decrease in renal clearance may also lead to an increase of these serum cytokines in cases of hemorrhage [21]. Hoke et al. [29] observed increased serum levels of IL-1*β*, IL-6, and IL-10 after bilateral nephrectomy. The increased extrarenal production, together with absence of renal elimination, explains the higher values of serum cytokines. Our results suggest that increased production of serum cytokines by extrarenal cells in cases of hemorrhage is considerable to the detriment of renal tissue production because the levels of these mediators in renal supernatant tissue did not differ, whether or not the group underwent renal hypoperfusion, although the renal tissue dosage may have been made too early, with insufficient time to detect any change in tissue levels of these mediators.

An enhanced formation of arachidonic acid metabolites by COX-2 contributes to the renal dysfunction caused by hemorrhagic shock in the rat [25]. In addition, increased inflammatory cytokines in ischemic renal injury play a important role in the pathophysiology of renal tissue injury [9]. Unfortunately, in this study, no dosage of prostanoids derived from arachidonic acid was performed. Therefore, further studies should be done to clarify whether the renal effects of parecoxib are due to inhibition of COX-2-derived prostanoids or due to inhibition of inflammatory mediators such as cytokines.

Renal medulla is the region of the kidney most vulnerable to ischemia. Any decrease in oxygen supply can cause tubular dysfunction and increased urinary sodium, activating the tubuloglomerular* feedback* mechanism in macula densa, with consequent arteriolar vasoconstriction and reduction in glomerular filtration [9, 23]. We observed that RBF and GFR were lower in the group undergoing hemorrhage pretreated with placebo. Although there was no statistical significance, rats pretreated with parecoxib showed better renal function after hemorrhage, with higher values of RBF and GFR compared to those receiving placebo.

The renal effects of parecoxib and other selective COX-2 inhibitors are controversial. Patel et al. [13] observed elevation of serum urea and creatinine levels, reduced creatinine* clearance,* and increased renal tissue damage in rats treated with parecoxib in an experimental model of ischemia-reperfusion. Other authors have also questioned renal safety of selective COX-2 inhibitors and disapproved its use in cases of renal hypoperfusion [14, 30]. However, several studies have shown that blockade of COX-2 is associated with improved outcomes in function and histology of various organs after ischemia-reperfusion injury [22, 25, 31, 32]. Studies have shown that pretreatment with COX inhibitors, selective or not, improved renal function and decreased levels of renal fibrosis and tubular necrosis after renal injury [19, 22, 33]. Other authors have also detected improvement in renal function after use of parecoxib in renal injury [34, 35]. Despite these studies and our results showing beneficial effects of COX-2 inhibitors on renal injury after ischemia, further studies are needed to identify for how long and under what conditions beneficial effects are maintained and to determine the long-term renal effects of this drug class.

Whether selective COX-2 inhibitors alter renal vascular resistance is still controversial. Some studies show the effects of renal vasoconstriction when they are used for long periods, while others do not observe changes in renal hemodynamics, either short term or long term [12, 36, 37].

Hypovolemia causes an increase in renal vascular resistance and decreases RBF and GFR, according to the tubuloglomerular* feedback* mechanism, observed in hemorrhage group pretreated with placebo. Heller and Horáček [38] observed earlier and more pronounced decrease in RBF and GFR after hypotension by hemorrhage than after clamping of the renal artery. They also observed that the sharp decline in RBF during hemorrhage was accompanied by a significant increase in RVR, from both the afferent and efferent arterioles. Likewise, in this study, we found that Plc/H group showed greater RVR than the Plc/NH group. Although there was no statistical significance, the hemorrhage group pretreated with parecoxib showed lower RVR after hemorrhage, compared to that pretreated with placebo. The use of this selective COX-2 inhibitor may have contributed to inhibiting compensatory vasoconstriction resulting from reduction in RBF, preventing the increase in RVR in such cases.

The COX-2 enzyme in the macula densa regulates tubuloglomerular* feedback*, suppresses nitric oxide (NO), and enhances the effects of TXA2. Inhibition of COX-2 by parecoxib suppresses tubuloglomerular* feedback*, increasing the NO/TXA2 ratio and inhibiting renal vasoconstriction [24]. Then parecoxib can inhibit renal vasoconstriction in rats subjected to hemorrhage, whose levels of RVR were similar to those of rats not subjected to hemorrhage. By inhibiting tubuloglomerular* feedback*, parecoxib could allow greater glomerular blood flow and greater GFR in the group undergoing renal hypoperfusion.

Vascular congestion and ectasia occurred similarly in the kidneys in all studied groups. There was no leukocyte infiltration in any of the groups. The hemorrhage group pretreated with placebo presented a higher percentage of tubular dilation and degeneration if compared to the group that received parecoxib, indicating that previous use of parecoxib prevented tubular injuries in this situation. Likewise, Guedes et al. [39] observed a lower degree of acute tubular necrosis in rats subjected to acute hemorrhage and pretreated with nonselective NSAID (ketoprofen). Our findings corroborate the protective effects of parecoxib against renal tubular injury of hypoperfusion.

Treatment with selective COX-2 inhibitor also prevented tubular lesions and reduced interstitial fibrosis in kidney undergoing ureteral obstruction, as COX-2 triggers secretion of TGF-*β*, leading to collagen and fibrin deposition in the affected kidney [40]. Cheng and Harris [41] suggests that long-term use of selective COX-2 inhibitors exhibits potential renoprotective effects in cases of glomerular hyperfiltration, such as those occurring in diabetes and postpartial nephrectomy status, and reduces proteinuria, deposition of extracellular matrix, and glomerular sclerosis in these cases. Lower scores of tubular degeneration in kidneys of rats subjected to hemorrhage pretreated with parecoxib indicate the beneficial effects of using a single dose of this selective COX-2 inhibitor in the prevention of ischemic tubular injury caused by hemorrhagic shock.

## 5. Conclusion

Pretreatment with a single dose of parecoxib reduced inflammatory response and histological renal injury, without altering renal function in rats subjected to acute hemorrhage. However, these short-term effects may not represent benefit of this drug in the long term. The use of this selective COX-2 inhibitor, a drug widely used to prevent and treat acute pain in large surgeries, remains controversial in situations of hemorrhagic shock.

## Figures and Tables

**Table 1:** Serum cytokine levels in each of the four groups of rats.

Parameter	Plc/NH (*n* = 11)	Pcx/NH (*n* = 11)	Plc/H (*n* = 11)	Pcx/H (*n* = 11)
IL-1*α*	36.8 ± 12.3	27.9 ± 16.1	60.2 ± 40.6^*∗*^	37.0 ± 17.9
IL-1*β*	41.5 ± 19.0	50.5 ± 41.1	66.0 ± 21.0^*∗*^	48.6 ± 23.4
IL-6	142.9 ± 69.3	205.1 ± 193.3	449.2 ± 160.8^*∗*^	229.3 ± 241.9
IL-10	123.5 ± 103.2	58.0 ± 30.7	365.3 ± 322.1^*∗*^	182.6 ± 99.9
TNF-*α*	21.2 ± 7.7	20.4 ± 18.6	42.4 ± 23.2^*∗*^	27.1 ± 12.6

Data were expressed as picogram per mL of serum; values are means ± SD; *n* is number of animals. Plc/NH = placebo/no hemorrhage group, Pcx/NH = parecoxib/no hemorrhage group, Plc/H = Placebo/hemorrhage group, and Pcx/H = parecoxib/hemorrhage group; ^*∗*^Plc/H > Plc/NH, Pcx/NH, and Pcx/H, *p* < 0.05.

**Table 2:** Cytokine levels in the supernatant renal tissue in each of the four groups of rats.

Parameter	Plc/NH (*n* = 11)	Pcx/NH (*n* = 11)	Plc/H (*n* = 11)	Pcx/H (*n* = 11)
IL-1*α*	17.3 ± 4.2	16.7 ± 3.7	14.2 ± 4.1	17.3 ± 6.1
IL-1*β*	3.7 ± 1.2	3.7 ± 1.1	4.0 ± 0.6	3.8 ± 1.3
IL-6	44.6 ± 17.6	42.9 ± 15.8	43.5 ± 11.4	44.8 ± 20.3
IL-10	129.7 ± 37.5	131.1 ± 32.0	142.4 ± 23.0	137.8 ± 48.7
TNF-*α*	9.1 ± 3.9	8.8 ± 4.5	9.3 ± 3.0	10.0 ± 5.6

Data were expressed as picogram per milligram of protein. Values are means ± SD; *n* is number of animals. Plc/NH = placebo/no hemorrhage group, Pcx/NH = parecoxib/no hemorrhage group, Plc/H = placebo/hemorrhage group, and Pcx/H = parecoxib/hemorrhage group; *p* > 0.05.

**Table 3:** Renal function values in each of the four groups of rats.

Parameter	Plc/NH (*n* = 11)	Pcx/NH (*n* = 11)	Plc/H (*n* = 11)	Pcx/H (*n* = 11)
*C*_PAH_ (mL·min^−1^)^†^	0.018 ± 0.006	0.014 ± 0.006	0.008 ± 0.002	0.011 ± 0.003
*C*_IOT_ (mL·min^−1^)^‡^	0.003 ± 0.001	0.004 ± 0.001	0.002 ± 0.001	0.003 ± 0.001
FF	0.211 ± 0.135	0.320 ± 0.125	0.294 ± 0.192	0.294 ± 0.178
RVR (mmHg·min^−1^·mL^−1^)^#^	4.906 ± 3.200	6.999 ± 2.680	12.027 ± 6.855	6.588 ± 3.070

Values are means ± SD; *n* is number of animals. *C*_PAH_ = clearance of sodium para-aminohippurate, *C*_IOT_ = clearance of sodium iothalamate, FF = filtration fraction, RVR = renal vascular resistance, Plc/NH = placebo/no hemorrhage group, Pcx/NH = parecoxib/no hemorrhage group, Plc/H = placebo/hemorrhage group, and Pcx/H = parecoxib/hemorrhage group; ^†^Plc/NH > Plc/H and Pcx/H, *p* < 0.001 and Pcx/NH > Plc/H, *p* < 0.001; ^‡^Pcx/NH > Plc/H, *p* < 0.001; ^#^Plc/H > Plc/NH, *p* < 0.05.

## References

[1] Gilron I., Milne B., Hong M. (2003). Cyclooxygenase-2 Inhibitors in Postoperative Pain Management: Current Evidence and Future Directions. *Anesthesiology*.

[2] Langford R. M., Joshi G. P., Gan T. J. (2009). Reduction in opioid-related adverse events and improvement in function with parecoxib followed by valdecoxib treatment after non-cardiac surgery: a randomized, double-blind, placebo-controlled, parallel-group trial. *Clinical Drug Investigation*.

[3] Nussmeier N. A., Whelton A. A., Brown M. T. (2006). Safety and efficacy of the cyclooxygenase-2 inhibitors parecoxib and valdecoxib after noncardiac surgery. *Anesthesiology*.

[4] Zhang J., Ding E. L., Song Y. (2006). Adverse effects of cyclooxygenase 2 inhibitors on renal and arrhythmia events: meta-analysis of randomized trials. *The Journal of the American Medical Association*.

[5] Lloyd R., Derry S., Moore R. A., McQuay H. J. (2009). Intravenous or intramuscular parecoxib for acute postoperative pain in adults.. *Cochrane Database of Systematic Reviews (Online)*.

[6] Malan T. P., Marsh G., Hakki S. I., Grossman E., Traylor L., Hubbard R. C. (2003). Parecoxib sodium, a parenteral cyclooxygenase 2 selective inhibitor, improves morphine analgesia and is opioid-sparing following total hip arthroplasty. *Anesthesiology*.

[7] Niruthisard S., Werawataganon T., Bunburaphong P., Ussawanophakiat M., Wongsakornchaikul C., Toleb K. (2007). Improving the analgesic efficacy of intrathecal morphine with parecoxib after total abdominal hysterectomy. *Anesthesia & Analgesia*.

[8] Akcay A., Nguyen Q., Edelstein C. L. (2009). Mediators of inflammation in acute kidney injury. *Mediators of Inflammation*.

[9] Bonventre J. V., Zuk A. (2004). Ischemic acute renal failure: an inflammatory disease?. *Kidney International*.

[10] Kielar M. L., John R., Bennett M. (2005). Maladaptive role of IL-6 in ischemic acute renal failure. *Journal of the American Society of Nephrology*.

[11] Cassatella M. A., Meda L., Bonora S., Ceska M., Constantin G. (1993). Interleukin 10 (IL-10) inhibits the release of proinflammatory cytokines from human polymorphonuclear leukocytes. Evidence for an autocrine role of tumor necrosis factor and IL-1*β* in mediating the production of IL-8 triggered by lipopolysaccharide. *The Journal of Experimental Medicine*.

[12] Cheng H. F., Harris R. C. (2005). Renal effects of non-steroidal anti-inflammatory drugs and selective cyclooxygenase-2 inhibitors. *Current Pharmaceutical Design*.

[13] Patel N. S. A., Cuzzocrea S., Collino M. (2007). The role of cycloxygenase-2 in the rodent kidney following ischaemia/reperfusion injury *in vivo*. *European Journal of Pharmacology*.

[14] Perazella M. A., Eras J. (2000). Are selective COX-2 inhibitors nephrotoxic?. *American Journal of Kidney Diseases*.

[15] Diego L. A. D. S., Marques C. D., Vianna P. T. G., Viero R. M., Braz J. R. C., Castiglia Y. M. M. (2007). Glibenclamide effects on renal function and histology after acute hemorrhage in rats under sevoflurane anesthesia. *Renal Failure*.

[16] Silva M. D. S., Machado Castiglia Y. M., Galvão Vianna P. T., Viero R. M., Cerqueira Braz J. R., Cassetari M. L. (2006). Rat model of depending prostaglandin renal state: Effect of ketoprofen. *Renal Failure*.

[17] Rönnhedh C., Jaquenod M., Mather L. E. (1996). Urineless estimation of glomerular filtration rate and renal plasma flow in the rat. *Journal of Pharmacological and Toxicological Methods*.

[18] Patel N. S. A., Chatterjee P. K., Di Paola R. (2005). Endogenous interleukin-6 enhances the renal injury, dysfunction, and inflammation caused by ischemia/reperfusion. *The Journal of Pharmacology and Experimental Therapeutics*.

[19] Feitoza C. Q., Goncalves G. M., Semedo P., Cenedeze M. A., Pinheiro H. S., Beraldo F. C. (2008). Inhibition of COX 1 and 2 prior to renal ischemia/reperfusion injury decreases the development of fibrosis. *Molecular Medicine*.

[20] Donnahoo K. K., Shames B. D., Harken A. H., Meldrum D. R. (1999). The role of tumor necrosis factor in renal ischemia-reperfusion injury. *The Journal of Urology*.

[21] Andres-Hernando A., Dursun B., Altmann C. (2012). Cytokine production increases and cytokine clearance decreases in mice with bilateral nephrectomy. *Nephrology Dialysis Transplantation *.

[22] Feitoza C. Q., Câmara N. O. S., Pinheiro H. S. (2005). Cyclooxygenase 1 and/or 2 blockade ameliorates the renal tissue damage triggered by ischemia and reperfusion injury. *International Immunopharmacology*.

[23] Brezis M., Rosen S. (1995). Hypoxia of the renal medulla—its implications for disease. *The New England Journal of Medicine*.

[24] Araujo M., Welch W. J. (2009). Cyclooxygenase 2 inhibition suppresses tubuloglomerular feedback: Roles of thromboxane receptors and nitric oxide. *American Journal of Physiology-Renal Physiology*.

[25] McDonald M. C., Mota-Filipe H., Paul A. (2001). Calpain inhibitor I reduces the activition of nuclear factor-*κ*B and organ injury/dysfunction in hemmorrhagic shock. *The FASEB Journal*.

[26] Feitoza C. Q., Semedo P., Gonçalves G. M. (2010). Modulation of inflammatory response by selective inhibition of cyclooxygenase-1 and cyclooxygenase-2 in acute kidney injury. *Inflammation Research*.

[27] Kurcer Z., Oguz E., Ozbilge H. (2007). Melatonin protects from ischemia/reperfusion-induced renal injury in rats: this effect is not mediated by proinflammatory cytokines. *Journal of Pineal Research*.

[28] Donnahoo K. K., Meng X., Ayala A., Cain M. P., Harken A. H., Meldrum D. R. (1999). Early kidney TNF-*α* expression mediates neutrophil infiltration and injury after renal ischemia-reperfusion. *American Journal of Physiology-Regulatory, Integrative and Comparative Physiology*.

[29] Hoke T. S., Douglas I. S., Klein C. L. (2007). Acute renal failure after bilateral nephrectomy is associated with cytokine-mediated pulmonary injury. *Journal of the American Society of Nephrology*.

[30] Breyer M. D., Hao C.-M., Qi Z. (2001). Cyclooxygenase-2 selective inhibitors and the kidney. *Current Opinion in Critical Care*.

[31] Candelario-Jalil E., González-Falcón A., García-Cabrera M., León O. S., Fiebich B. L. (2007). Post-ischaemic treatment with the cyclooxygenase-2 inhibitor nimesulide reduces blood-brain barrier disruption and leukocyte infiltration following transient focal cerebral ischaemia in rats. *Journal of Neurochemistry*.

[32] Hamada T., Tsuchihashi S., Avanesyan A. (2008). Cyclooxygenase-2 deficiency enhances Th2 immune responses and impairs neutrophil recruitment in hepatic ischemia/reperfusion injury. *The Journal of Immunology*.

[33] Feitoza C. Q., Sanders H., Cenedeze M., Câmara N. O. S., Pacheco-Silva A. (2002). Pretreatment with indomethacin protects from acute renal failure following ischemia-reperfusion injury. *Transplantation Proceedings*.

[34] Höcherl K., Schmidt C., Bucher M. (2009). COX-2 inhibition attenuates endotoxin-induced downregulation of organic anion transporters in the rat renal cortex. *Kidney International*.

[35] Hauser B., Fröba G., Bracht H. (2005). Effects of intrarenal administration of the cox-2 inhibitor parecoxib during porcine suprarenal aortic cross-clamping. *Shock*.

[36] Harding P., Carretero O. A., Beierwaltes W. H. (2000). Chronic cyclooxygenase-2 inhibition blunts low sodium-stimulated renin without changing renal haemodynamics. *Journal of Hypertension*.

[37] López R., Roig F., Llinás M. T., Salazar F. J. (2003). Role of cyclooxygenase-2 in the control of renal haemodynamics and excretory function. *Acta Physiologica Scandinavica*.

[38] Heller J., Horáček V. (1997). Glomerular haemodynamics during renal artery clamping and haemorrhage in the dog. *Experimental Physiology*.

[39] Guedes F. S., da Cruz D. S., Rodrigues M. M. P. (2012). Renal histology and immunohistochemistry after acute hemorrhage in rats under sevofurane and ketoprofen effect. *Acta Cirurgica Brasileira*.

[40] Miyajima A., Ito K., Asano T., Seta K., Ueda A., Hayakawa M. (2001). Does cyclooxygenase-2 inhibitor prevent renal tissue damage in unilateral ureteral obstruction?. *The Journal of Urology*.

[41] Cheng H.-F., Harris R. C. (2004). Cyclooxygenases, the Kidney, and Hypertension. *Hypertension*.

